# SAFE (Sarpogrelate Anplone in Femoro-popliteal artery intervention Efficacy) study: study protocol for a randomized controlled trial

**DOI:** 10.1186/s13063-017-2155-5

**Published:** 2017-09-22

**Authors:** Sanghyun Ahn, Joongyub Lee, Seung-Kee Min, Jongwon Ha, Sang-il Min, Song-Yi Kim, Min-Ji Cho, Sungsin Cho, SAFE study investigators

**Affiliations:** 1 Department of Surgery, Seoul National University Hospital, 101 Daehak-ro, Jongno-gu, Seoul, 03080 South Korea; 2 Division of Clinical Epidemiology, Medical Research Collaborating Center, Biomedical Research Institution, Seoul National University Hospital, Seoul, South Korea

**Keywords:** Endovascular treatment, Sarpogrelate, Peripheral artery disease, Stenosis, Femoro-popliteal

## Abstract

**Background:**

Sarpogrelate is expected to reduce restenosis by protecting blood vessels from oxidative stress and vascular endothelial dysfunction as well as by acting as an antiplatelet agent after endovascular treatment (EVT). This trial was designed to compare aspirin plus sustained-release (SR) sarpogrelate with aspirin plus clopidogrel for the prevention of restenosis in patients with femoro-popliteal (FP) peripheral artery disease (PAD) who underwent EVT.

**Methods/Design:**

This is an open label, multicenter, prospective randomized controlled clinical trial. Patients will be eligible for inclusion in this study if they require EVT for stenosis or occlusion of a de novo FP lesion. Patients in each group will receive aspirin 100 mg with clopidogrel 75 mg or aspirin 100 mg with SR sarpogrelate 300 mg (Anplone®) orally once a day for six months. The primary outcome of the study is the restenosis rate, defined as > 50% luminal reduction by computed tomography angiography or catheter angiography in the six-month follow-up period. Secondary outcomes include target lesion revascularization, major bleeding, ipsilateral major amputation, all-cause mortality, and all adverse events that take place in those six months.

**Discussion:**

This study is a multicenter randomized controlled trial designed to show non-inferiority in terms of the re-stenosis rate of SR sarpogrelate compared to clopidogrel for EVT for PAD in FP lesion patients.

**Trial registration:**

ClinicalTrials.gov, NCT02959606. Registered on 9 November 2016.

**Electronic supplementary material:**

The online version of this article (doi:10.1186/s13063-017-2155-5) contains supplementary material, which is available to authorized users.

## Background

After endovascular treatment (EVT) for peripheral artery disease (PAD), antiplatelet agents are used for three purposes: to prevent early thrombosis; to maintain long-term patency; and to reduce the incidence of cardiovascular events. Aspirin-based monotherapy or dual antiplatelet therapy (DAPT) are the most commonly used methods for these purposes [1]. Even though the common guidelines for PAD suggest mono-antiplatelet therapy, monotherapy alone may not be enough [2–5]. Surprisingly, however, there is no evidence-based recommendation for optimal antiplatelet therapy after EVT in PAD [6]. DAPT consisting of aspirin and clopidogrel, which is widely used, is only based on several cardiac-related studies [5].

However, even with the same antiplatelet agents, the effects may differ in cardiovascular disease (CAD) and PAD. For example, aspirin plus dipyridamol was effective in patients with CAD but not in those with PAD [7]. In addition, the incidence of not responding to clopidogrel was 30% higher in cases of EVT for PAD compared to percutaneous coronary intervention (PCI) [8]. Although the reasons for this phenomenon have not been uncovered, it may not be right to use the same DAPT in patients with PAD and CVD. Also, the role of antiplatelet therapy may be reduced or altered by technological developments in EVT. Therefore, an appropriate evidence-based recommendation of antiplatelet agents after EVT for PAD is needed.

Sarpogrelate is a selective 5-hydroxytryptamine receptor 2A antagonist that inhibits platelet aggregation, vasocontraction, and vascular smooth muscle proliferation [9]. Sarpogrelate is also expected to reduce restenosis by protecting blood vessels from oxidative stress and vascular endothelial dysfunction as well as by acting as an antiplatelet agent after EVT [10].

However, a commercially available immediate-release formulation of sarpogrelate (100 mg, three times per day) is not as convenient for patients to use as the currently used clopidogrel (75 mg, once daily). Fortunately, a sustained-release (SR) formulation of 300 mg sarpogrelate hydrochloride for once-daily usage has been introduced to improve convenience and compliance [11, 12].

This trial was designed to compare aspirin plus SR sarpogrelate with aspirin plus clopidogrel for the prevention of restenosis (>50% stenosis) at six-month follow-up in patients with femoro-popliteal (FP) PAD who underwent EVT.

## Methods/Design

### Study objectives, hypothesis, and design

**Fig 1:**
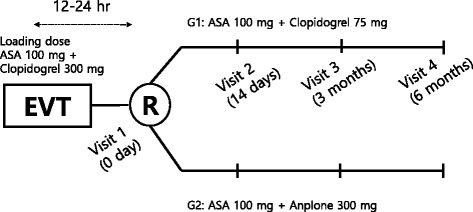
*Flow diagram* of SAFE study. *EVT* endovascular treatment, *ASA* aspirin, *R* randomization

**Fig. 2:**
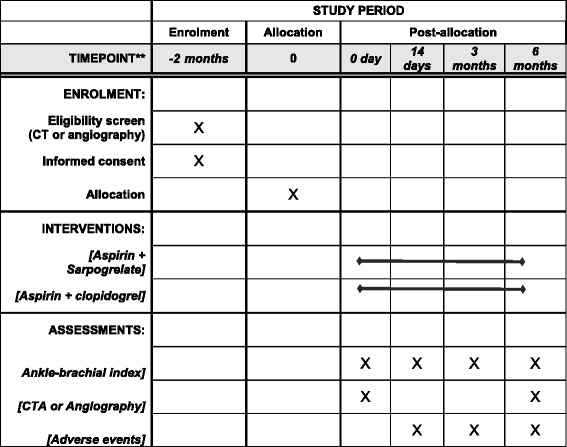
Standard Protocol Items: Recommendations for Interventional Trials (SPIRIT) figure (Additional file 1)

This is an open label, multicenter, prospective randomized controlled clinical trial that will evaluate the efficacy and safety of aspirin plus SR sarpogrelate in PAD patients undergoing EVT for FP lesions compared with aspirin plus clopidogrel (ClinicalTrials.gov, NCT02959606). After baseline measurements and EVT, participants will be randomly allocated into the aspirin plus clopidogrel group or the aspirin plus SR sarpogrelate group. A brief flowchart of the whole study is presented in Figs. 1 and 2.

### Study population and eligibility

Patients will be eligible for inclusion in this study if they require EVT for stenosis or occlusion of a de novo FP lesion. To be eligible for randomization, patients must have adequate inflow and outflow (with or without intervention of the iliac or below-the-knee arteries) and successful FP intervention (residual stenosis < 30%). Previous intervention for an FP lesion is an exclusion criterion. Other inclusion and exclusion criteria are listed in Table 1.

**Table 1:** Study eligibility criteria

*Inclusion criteria*	
Age > 18 years	
Angiographically confirmed significant FP stenosis or occlusion by atherosclerosis	
Successful FP intervention (residual stenosis < 30% after EVT)	
Without significant residual inflow disease; intact iliac artery inflow (with or without intervention of iliac or below knee arteries)	
Patent outflow status; at least one arterial runoff in BTK	
All kind of FP interventions including POBA, stent, DCB, DES for TASC A ~ D	
*Exclusion criteria*	
At risk of hemorrhage, bleeding tendency, or thrombophilia	
Acute limb ischemia / inflammatory arterial disease	
Contraindication or allergic to ASA, clopidogrel, Anplone	
Medication of warfarin	
Pregnancy, hepatic dysfunction, thrombocytopenia	
Previous FP bypass surgery or intervention	
Impossible to stop clopidogrel before EVT	
Unable to give informed consent.	

*FP* femoro-popliteal, *EVT* endovascular treatment, *BTK* below-the-knee, *POBA* plain old balloon angioplasty, *DCB* drug-coated balloon, *DES* drug-eluting stent, *TASC* The Trans-Atlantic Inter-Society Consensus, *ASA* aspirin

### Intervention and assessment schedule

All patients will be given a loading dose of 300 mg clopidogrel and 100 mg aspirin 24 hours or less before EVT. However, patients who have been treated with clopidogrel for > 5 days will receive 75 mg clopidogrel and 100 mg aspirin. Within 24 hours after successful FP intervention, the patients will be randomly assigned to the aspirin plus clopidogrel or aspirin plus SR sarpogrelate groups. Patients in each group will receive aspirin 100 mg with clopidogrel 75 mg or aspirin 100 mg with SR sarpogrelate 300 mg (Anplone®) orally once a day for six months.

### Follow-up

Outcomes will be recorded at 14 days and three and six months after intervention (Table 2). Information to be collected will include clinical and hemodynamic status, laboratory tests (complete blood cell count, liver function test, prothrombin time International Normalized Ratio), ankle–brachial index (ABI), adverse events, and other health problems and computed tomography angiography (CTA).

**Table 2:** Assessment schedule

Visit date	Visit 1	Visit 2	Visit 3	Visit 4
	0 day	14 days	3 months	6 months
Demographic data	○			
Medical/surgical history	○			
Concomitant diseases	○		○	○
Concomitant medication/treatment	○		○	○
Physical examination (vital signs)	○	○	○	○
Laboratory results (CBC, LFT, PT INR)	○	○	○	○
ABI	○	○	○	○
CTA or angiography	○			○
Adverse events		○	○	○

*CBC* complete blood cell count, *LFT* liver function test, *PT* prothrombin time International Normalized Ratio, *ABI* ankle–brachial index, *CTA* computed tomography angiography

### Outcome measures

The primary outcome of the study is the restenosis rate, defined as > 50% luminal reduction by CTA or catheter angiography in the six-month follow-up period. Secondary outcomes include target lesion revascularization, major bleeding, ipsilateral major amputation, all-cause mortality, and all adverse events that take place in those six months.

### Adverse effects

Recording of serious adverse events (SAE) will conform to the Good Clinical Practice standards and the Research Governance Framework 2005. Analysis of safety-related data will be performed with respect to frequency of SAE including all-cause death, stroke, myocardial infarction (MI), or revascularization, early thrombosis, major bleeding, and major amputation. MI is defined as symptoms of ischemia with electrocardiographic changes compatible with MI or cardiac markers at least twice the upper normal limit [13]. Stroke is defined as brain hemorrhage, transient ischemic attack, or cerebral infarction with focal or global neurological deficits with signs or symptoms confirmed by a neurologist on the basis of neuroimaging results [14]. Major bleeding is defined as a need for a blood transfusion (≥ 2 packs), surgical intervention, or hypotension requiring inotropic support (hemoglobin reduction ≥ 2 g/dL). GUSTO bleeding criteria will be used [15]. Major amputation as a SAE is define as unplanned above-the-ankle amputation. Early thrombosis is defined as any event that occurs within 30 days after EVT. SAEs must be reported by the attending physician to the principal investigator within 24 h after the SAE becomes known.

### Statistical considerations

The aim of this study is to show the non-inferiority of sarpogrelate to clopidogrel in preventing restenosis. From the previous literature, we found a six-month restenosis rate in patients on clopidogrel of 11% and a six-month restenosis rate in patients on sarpogrelate of 6% following EVT for FP artery intervention (7). The targeted significance level is 2.5%; the sarpogrelate group proportion is assumed to be 16% under the null hypothesis of inferiority. A sample size of 136 in each group will achieve 80% power to detect a non-inferiority margin difference between the group proportions of 5% assuming a dropout rate of 10%.

Randomization will be done by an independent statistical core at the Medical Research Collaborating Center of Seoul National University Hospital. Permuted block randomization will be used with blocks of sizes 4 or 6 to ensure a balance between the two treatment groups. We will use SAS 9.4 (SAS institute Inc., Cary, NC, USA) for sequence generation, which is to be stratified for: (1) presence of occlusion within the index lesion; (2) use of stent; (3) TASC classification “A or B” vs. “C or D;” and (4) presence of critical limb ischemia. Allocation of treatment will be done via a web-based randomization system, which returns the treatment group after the input of a participant’s study ID and the inclusion/exclusion criteria.

Characteristics of participants will be summarized using mean and standard deviation for continuous variables and frequency and percentage for categorical variables. A two-sided 95% confidence interval for a difference in restenosis rate between the two treatment groups will be calculated; then, whether the upper limit of the confidence interval should fall within the pre-determined margin of non-inferiority will be evaluated to prove the non-inferiority of sarpogrelate to clopidogrel. The conclusion will be based on the result of intention-to-treat analysis, while the per-protocol analysis result will be provided by utilizing data on patients with treatment compliance > 80%. Among the secondary outcomes, adverse events will be analyzed using chi-squared tests or Fisher’s exact tests as appropriate. Major bleeding, ipsilateral amputation, revascularization, and all-cause mortality will be compared using log-rank tests and survival curves constructed using Kaplan–Meier methods.

Significance tests will be two-sided. A *P* value of < 0.05 according to SAS will be taken to indicate a statistically significant difference between the groups. SAS 9.4 (SAS institute Inc., Cary, NC, USA) will be used for data analysis.

### Ethical approval

This study follows the principles of the Helsinki Declaration and all patients will provide written informed consent prior to participation. Participation can be withdrawn at any time without any negative consequences concerning current or future medical treatment. Study approval was given by the Institutional Review Board of Seoul National University Hospital (IRB No. 1607-151-778).

## Discussion

This study is designed to present evidence of the suitability of antiplatelet agents after endovascular revascularization in patients with FP arterial disease.

Some patients who participate in this study may have multi-level disease, including inflow or outflow problems as well as FP lesions, which may need to be corrected simultaneously. In other words, the inclusion criteria are broad, which reflects the situation in clinical practice. We will stratify the data on occlusion of the index lesion, use of stents, TASC AB and CD, and critical limb ischemia, which will help to determine the use of antiplatelet agent.

To date, DAPT, which consists of aspirin and clopidogrel, is well documented in patients with PCI. ESC guidelines recommend DAPT for one month after EVT with a stent for PAD [16]. However, the optimal antiplatelet agent is still unclear in long-term cases. The role of antiplatelet agents may decline after EVT for PAD owing to the development of new technology and devices that has occurred over the past decade [17]. Alternatively, the use of stronger antiplatelet agents may be required owing to various forces in the femoral artery during exercise and more aggressive treatment.

In the CAPRIE study, aspirin alone was associated with a higher rate of ischemic events than was clopidogrel [18]. That emphasized the necessity of DAPT rather than aspirin alone. Especially for cases of MI, ischemic stroke, and symptomatic PAD, DAPT with clopidogrel was more effective than ASA alone [19]. On the other hand, CASPAR failed to demonstrate a benefit of DAPT (aspirin plus clopidogrel), reminding us of the need for a variety of independent studies on the use of antiplatelet agents in PAD [20].

In one study, sarpogrelate reduced the restenosis rate (4.6% vs. 28.6%) after six months of coronary stenting [21]. There are three inhibitory effects of sarpogrelate: anti-platelet aggregation and anti-vasoconstriction prevent the development of early thrombus and anti-vascular smooth muscle proliferation is expected to reduce in-stent restenosis [9]. In addition, to protect endothelial cells by dose-dependently reducing the ICAM-1 level in a hyperglycemic state, it may also improve long-term patency after EVT [22].

Considering that patients with PAD are at high risk for bleeding, sarpogrelate has the potential benefit of lower bleeding risk compared to clopidogrel [23, 24]. In Korea, the price of sarpogrelate is lower than that of clopidogrel, which can reduce medical expenses (US$0.92 vs. US$1.02 per one tablet). Such potential benefits of sarpogrelate may be a good alternative if it is not inferior to clopidogrel-based DAPT. In conclusion, the SAFE study is a multicenter randomized controlled trial designed to show non-inferiority in terms of the re-stenosis rate of SR sarpogrelate compared to clopidogrel for EVT for PAD in FP lesion patients. We hope to address these issues in the SAFE study.

### Trial status

Recruiting is ongoing.

## Additional file

Additional file 1: SPIRIT 2013 Checklist: recommended items to address in a clinical trial protocol and related documents. (DOC 121 kb)

## Abbreviations

ABI: Ankle–brachial index
CTA: Computed tomography angiography
CVD: Cardiovascular disease
DAPT: Dual antiplatelet therapy
EVT: Endovascular treatment
FP: Femoro-popliteal
PAD: Peripheral artery disease
PCI: Percutaneous coronary intervention
TASC: Transatlantic Inter-Society Consensus
